# Expression of epithelial growth factor receptor as a protein marker in oral reticular and erosive lichen planus

**DOI:** 10.1186/s12903-024-04507-z

**Published:** 2024-06-24

**Authors:** Forooz Keshani, Neda Kargahi, Mohammad Hossein Nikbakht, Shekufe Najafi, Fateme Fallah

**Affiliations:** 1 Department of Oral and Maxillofacial Pathology, Dental Research Center, Dental Research Institute, School of Dentistry, Isfahan University of Medical Sciences, Isfahan, Iran; 2 Student Research committee, School of Dentistry, Isfahan University of Medical Sciences, Isfahan, Iran; 3 Dental Research Center, Department of Pediatric Dentistry, School of Dentistry, Isfahan University of Medical Sciences, Isfahan, Iran

**Keywords:** Epithelial growth factor receptor, Oral lichen planus, Immunohistochemistry, EGFR

## Abstract

**Background:**

Oral lichen planus (OLP) is a chronic inflammatory mucosal disease that is classified as a premalignant condition. Epithelial growth factor receptor (EGFR) is associated with tumorigenesis and tumor progression and is overexpressed in several oral malignant disorders. Despite the association of EGFR overexpression with oral potentially malignant lesions, few studies have analyzed its expression in OLP, showing controversial results. This study aimed to compare the expression of EGFR as a protein marker in Reticular and Erosive OLP.

**Methods:**

This descriptive-analytical cross-sectional was conducted on 15 paraffin blocks of reticular lichen planus lesions, 16 paraffin blocks of erosive OLP lesions, and 8 paraffin blocks of inflammatory fibrous hyperplasia lesions as the control group (39 in total). After immunohistochemical staining for EGFR, samples were simultaneously observed by two maxillofacial pathologist, and the percentage of stained cells, intensity of staining, pattern of staining, and the location of stained cells were obtained.

**Results:**

The Mann-Whitney-U test showed that there was no significant difference in the mean percentage of stained cells between erosive OLP and reticular OLP (*P*-value = 0.213) and between reticular OLP and control group (*P*-value = 0.137), but there was a significant difference between erosive OLP and control group (*P*-value = 0.035). Fisher’s exact test showed that there was no significant difference between the frequency distribution of staining patterns in three types of lesions (*P*-value = 0.90). Kruskal-Wallis test showed that there was no significant difference between the intensity of staining in the three groups (*P*-value = 0.19) and also there was no significant difference between the location of stained cells in different layers of the epithelium in the three groups (*P*-value = 0.90).

**Conclusions:**

The results of this study showed that in comparison of reticular OLP, erosive OLP, and the control group there was a significant difference just between erosive OLP and control group in the percentage of stained cells.

## Background

Oral lichen planus (OLP) is a chronic inflammatory mucosal disease with autoimmune nature affecting the buccal mucosa, tongue, and gingiva with an incidence of 0.5–2% of the general population [1–3]. Epithelial thinning and hyperkeratosis with serrated rete ridges and also hydropic degeneration of basal epithelial cells with infiltrating band-like lymphocytes (predominantly T-cells) can be seen in histological Sect [4]. This disease is typically characterized by the presence of white lace-like lesions, with or without atrophic or erosive areas [3].

OLP is classified as a potential premalignant condition [5] with a 0.44–1.2% malignant transformation rate [6]. The most dangerous consequence of this lesion is the development of oral squamous cell carcinoma [7]. OLP can be divided into six clinical subtypes: reticular, plaque-like, atrophic, erosive/ulcerative, popular, and bullous. Reticular, erosive, and plaque-like are the most common subtypes [8, 9]. The reticular form is the most common type of OLP, which is completely recognizable due to white and slightly raised lines with erythematous borders that are stretched in different directions and create a network-like appearance (Wickham’s lines). The erosive form is the second most common form of OLP that causes ulcers. White radial lines can often be seen in the peripheral parts of these ulcers. Patients with erosive OLP experience a wide range of discomforts, from burning to severe pain that may even interfere with their eating [10].

The probability of progressing to malignancy of erosive, atrophic, and plaque-like types is higher than other types of lesions, and the lesions in the tongue and buccal mucosa are more likely to progress to malignancy [11]. Atrophied oral mucosa in severe erosive OLP lesions has the highest risk of progressing to malignancy [12].

Epithelial growth factor receptor (EGFR) is a polypeptide containing 53 amino acids that are encoded by a gene on the short arm of chromosome 7 and belong to the human epithelial receptor (HER) growth factor family of tyrosine kinase receptors. Abnormalities of EGFR are widely associated with tumorigenesis and tumor progression [13]. Increased expression of the EGFR, as an intramembrane receptor, is associated with the occurrence of many cancers, including breast cancer, prostate cancer, and oral squamous cell carcinoma (OSCC) [14]. Due to the important role of this receptor in signaling for the proliferation, differentiation, and migration of all types of cells, it is effective as a mitogen in maintaining the integrity of epithelial cells and on the other hand in carcinogenesis [15].

Despite its great importance, the etiology of OLP has not yet been fully identified [16].

Several studies have investigated the role of EGFR in the pathogenesis of oral carcinoma. EGFR overexpression, which promotes the proliferation and differentiation of keratinocytes, is present in approximately 80% of OSCC [17].

Ma et al. [18] in 2022 described that EGFR is one of the most important targets in the development of OLP. Some studies showed overexpression of EGFR in OSCC [19]. The other study showed a progressive increase in EGFR expression, which was proportional to the severity of premalignant lesions [20]. Despite the association of EGFR overexpression with oral carcinogenesis of oral potentially malignant lesions, few studies have analyzed its expression in OLP, showing controversial results. One of these studies described low EGFR expression in OLP samples [21], but another study observed a high expression in all their samples of OLP [22]. In the study by Agha-Hosseini et al. [23], there was no significant difference in the level of EGFR between the saliva and serum of patients with OLP and patients with OSCC. Boccellino et al. [24] in 2023 developed a diagnostic test kit to predict the development of oral cancer based on the expression of EGFR and steroid receptors. They reported that this test is non-invasive, particularly reliable, very fast, and economical. Therefore, studies in this field can be the basis for the invention of effective methods that can improve the prognosis of the lesions by their early detection.

This study aimed to compare the expression of EGFR as a protein marker in Reticular and Erosive OLP.

## Method

### Ethical approval and study design

This descriptive-analytical cross-sectional study was approved by the Research Ethics Committee of Isfahan University of medical sciences (IR.MUI.REC.1396.3.401).

### Participants

The study was conducted on 20 paraffin blocks of reticular OLP lesions, 20 paraffin blocks of erosive OLP lesions (the samples did not have dysplasia), and 10 paraffin blocks of inflammatory fibrous hyperplasia lesions (which is an inflammatory and benign lesion as a control group) from patients who referred to the pathology department of the Faculty of Dentistry of Isfahan University of Medical Sciences in 2006–2016 (50 in total). Their lesions were accurately diagnosed based on clinicopathology criteria by two maxillofacial pathologists, simultaneously.

These diagnose were based on the American Academy of Oral and Maxillofacial Pathology approach published in 2016 [25]. Histopathological criteria include band-like or patchy, predominately lymphocytic infiltrate in the lamina propria confined to the epithelium-lamina propria interface, basal cell liquefactive (hydropic) degeneration, lymphocytic exocytosis, absence of epithelial dysplasia, and absence of verrucous epithelial architectural change.

Distorted paraffin blocks that do not have enough tissue and blocks on which immunohistochemical staining was not possible for any reason such as samples in which antigens of interest were masked or destroyed during the fixation process or the antibodies used do not recognize the target antigen were excluded from the study. Among the samples, five of them from the reticular group, four of them from the erosive group, and two from the control group were excluded from the study process. Finally, the analyzes were performed based on data from 39 samples.

### Setting

First of all, all specimens, which were stained by hematoxylin and eosin, were examined by two oral and maxillofacial pathologists, simulataneously. After confirming the diagnosis of samples, immunohistochemical staining for EGFR was carried out by streptavidin-biotin method with appropriate positive, negative, and reagent controls. The tissue sections were kept at 37 °C and fixed overnight at 600 °C before immunohistochemistry. Dewaxing was carried out in xylene and rehydration was carried out in gradient alcohol (absolute alcohol of 70% and 50%) and finally in distilled water for 5 min each. Blocking was carried out by using 3% H2O2 in methanol for 30 min. Antigen retrieval was carried out using citrate buffer (pH = 6.0) method to optimize staining for 120 min at 98 °C. The sections were immunostained with primary polyclonal antibody for EGFR (Scytek, USA). Sections were incubated overnight at 4 °C with primary antibody in a humid chamber. The following day, the sections were stained using labeled streptavidinbiotin biogenex kit (DAKO LSAB + system, K0679) with modified timings, and the sections were incubated for 2 h in the corresponding biotinylated secondary antibody solution, followed by conjugated streptavidin horseradish peroxidase complex for 1 h. Bound peroxidase was revealed using 0.05% 3- diaminobenzedinetetrahydro (DAB) in TBS. The sections were dehydrated, cleared and mounted [26].

Then the samples were simultaneously observed by two oral and maxillofacial pathologists with an optical microscope (Olympus/Tokyo) in a magnification of 400 in five non-overlapping fields. The slides were examined in terms of the percentage of stained cells, intensity of staining, the pattern of staining, and the location of stained cells.

### Data measurement

For the percentage of stained cells two oral and maxillofacial pathologists, simultaneously counted the stained cells and calculated the mean percentage of stained cells.

The pattern of staining was categorized into membranous, cytoplasmic, and membranous-cytoplasmic groups [27].

The intensity of staining was also evaluated as follows: very weak: hardly visible with 400 magnification, weak: easily seen with 400 magnification, weak to moderate: hardly seen at 100 magnification, moderate to severe: easily seen at 100 magnification, severe: seen at 40 magnification [26].

The location of stained cells was categorized into basal-parabasal, basal-parabasal-intermediate, upper intermediate, intermediate, and all layers groups.

### Study size

Samples were selected by easy sampling method. The following formula was used to determine the sample size in each group, assuming the number of samples in each group was equal (α = 0.05, (power of test) 1- β = 0.80, d = 15).

$$n=\frac{{\left({Z}_{1-\frac{\alpha }{2}}+{Z}_{1-\beta }\right)}^{2}({\sigma }_{1}^{2}+{\sigma }_{2}^{2})}{{d}^{2}}$$

In this study, 50 samples were used (20 for each group and 10 for the control group).

### Statistical methods

Data were analyzed in SPSS software version 20. The data were analyzed by descriptive statistical methods and Kruskal-Wallis, Man-Whitney-U, and Fisher’s exact tests. The significance level was considered α = 0.05.

## Results

The sample consisted of 39 paraffin blocks which were in three groups of reticular OLP, erosive OLP, and control with the number of 15, 16, and eight, respectively.

### Percentage of stained cells

**Fig. 1:**
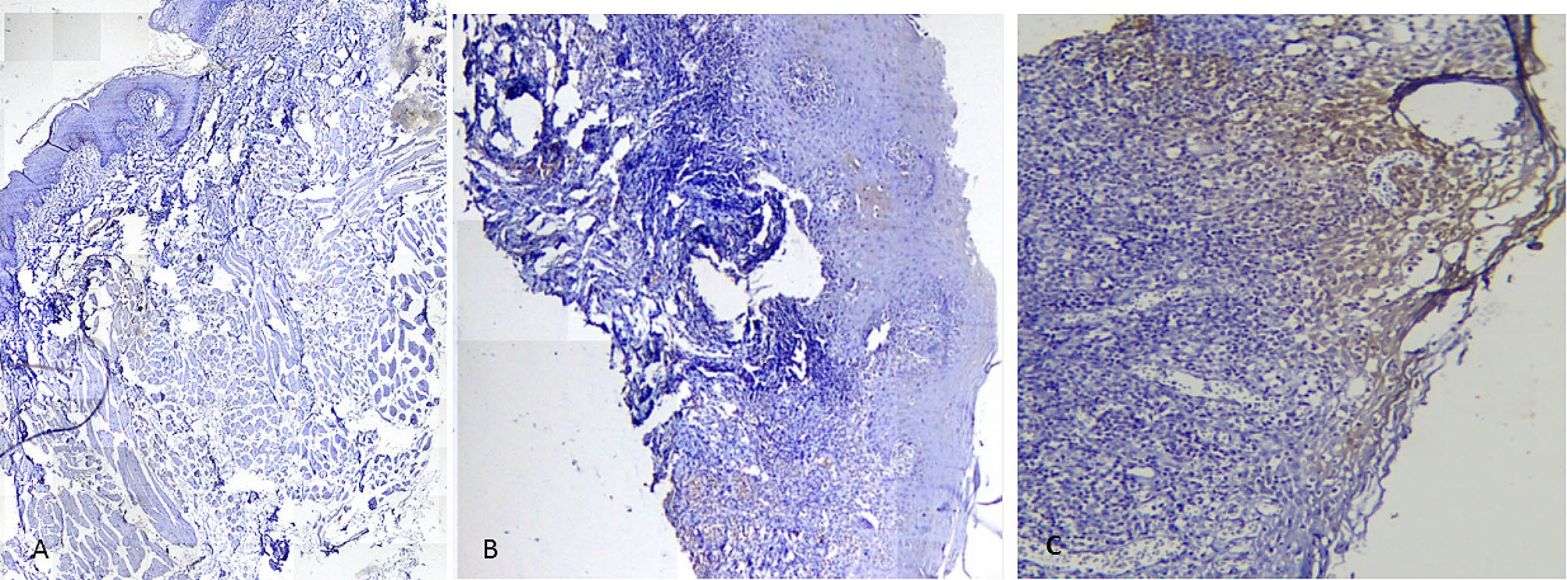
Photomicrograph shows, (**A**) Staining in Control group (400). (**B**) Staining in Reticular group (400). (**C**) Staining in Erosive group (400)

The mean percentage of stained cells in the first group (reticular) was 12.72 ± 7.30, in the second group (erosive) was 19.07 ± 13.58, in the third group (control) was 8.30 ± 4.77 (Fig. 1).

The Mann-Whitney-U test showed that there was no significant difference in the mean percentage of stained cells between erosive OLP and reticular OLP (*P*-value = 0.213) and between reticular OLP and control group (*P*-value = 0.137), but there was a significant difference between erosive OLP and control group (*P*-value = 0.035).

### Pattern of staining

**Fig. 2:**
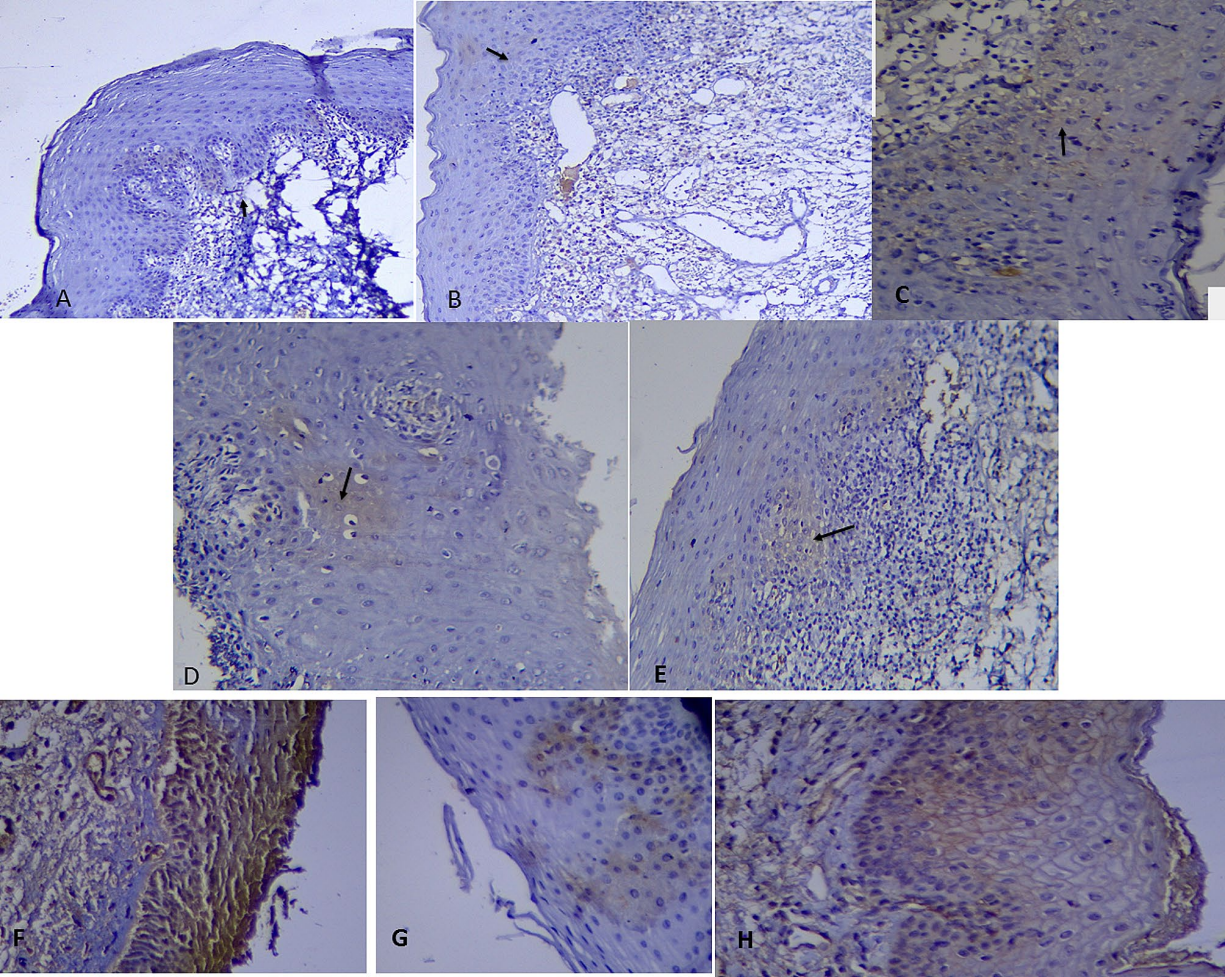
Photomicrograph shows, (**A**) Staining with cytoplasmic pattern in control group (100). (**B**) Staining with cytoplasmic-membranouse pattern in erosive group (100). (**C**) Staining with membranous pattern in reticular group (400). (**D**) Staining with cytoplasmic pattern in reticular group (200). (**E**) Staining with cytoplasmic pattern in erosive group (200). (**F**) Staining with cytoplasmic-membranouse pattern in erosive group (400). (**G**) Staining with cytoplasmic pattern (400). (**H**) Staining with membranous pattern in reticular group (400)

The number and percentage of samples in different groups with various staining patterns showed in Table 1. Fisher’s exact test showed that there was no significant difference between the frequency distribution of staining patterns in 3 types of lesions (*P*-value = 0.90) (Fig. 2).

**Table 1:** Pattern of staining in three groups

Pattern of staining		Reticular OLP (*n* = 15)	Erosive OLP (*n* = 16)	Control (*n* = 8)	*P*-Value
membranous	number percentage	1 6.67	0 0	0 0	0.90
cytoplasmic	number percentage	14 93.33	15 93.75	8 100.00	
membranous-cytoplasmic	number percentage	0 0	1 6.25	0 0	

### Intensity of staining

**Fig. 3:**
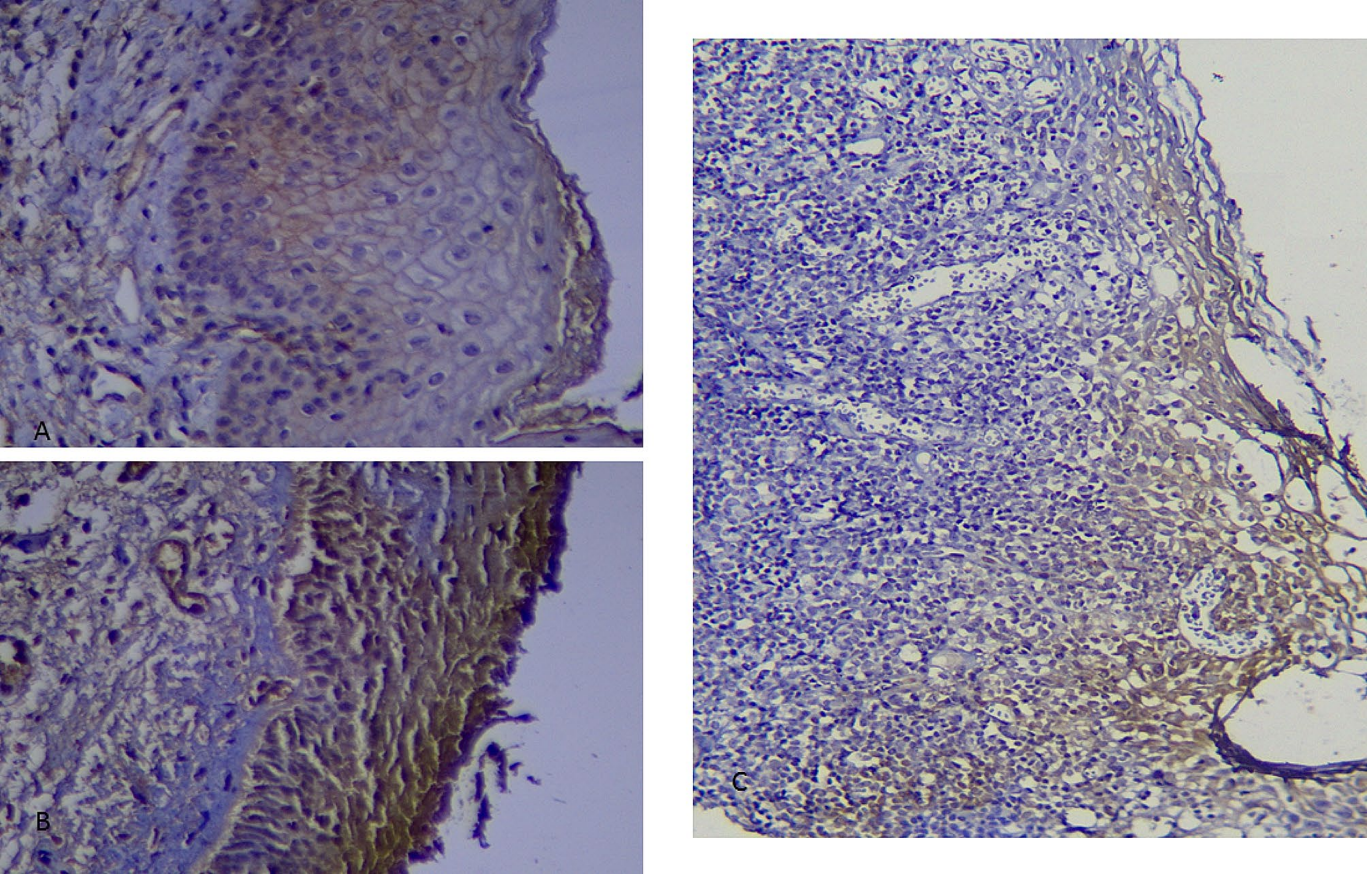
Photomicrograph shows, (**A**) Staining with moderate to severe intensity in reticular group (400). (**B**) Staining with sever intensity in erosive group (400). (**C**) Staining with sever intensity in erosive group (200)

The number and percentage of samples in different groups of staining Intensity showed in Table 2. Kruskal-Wallis test showed that there was no significant difference between the intensity of staining in 3 groups (*P*-value = 0.19) (Fig. 3).

**Table 2:** Intensity of staining in three groups

Intensity of staining		Reticular OLP (*n* = 15)	Erosive OLP (*n* = 16)	Control (*n* = 8)	*P*-Value
Very weak	number percentage	2 13.33	1 6.25	0 0	0.19
Weak	number percentage	5 33.33	2 12.50	2 25.00	
Weak to Moderate	number percentage	3 20.00	5 31.25	1 12.50	
Moderate to Severe	number percentage	5 33.33	5 31.25	5 62.50	
Severe	number percentage	0 0	3 18.75	0 0	

### Location of stained cells

**Fig. 4:**
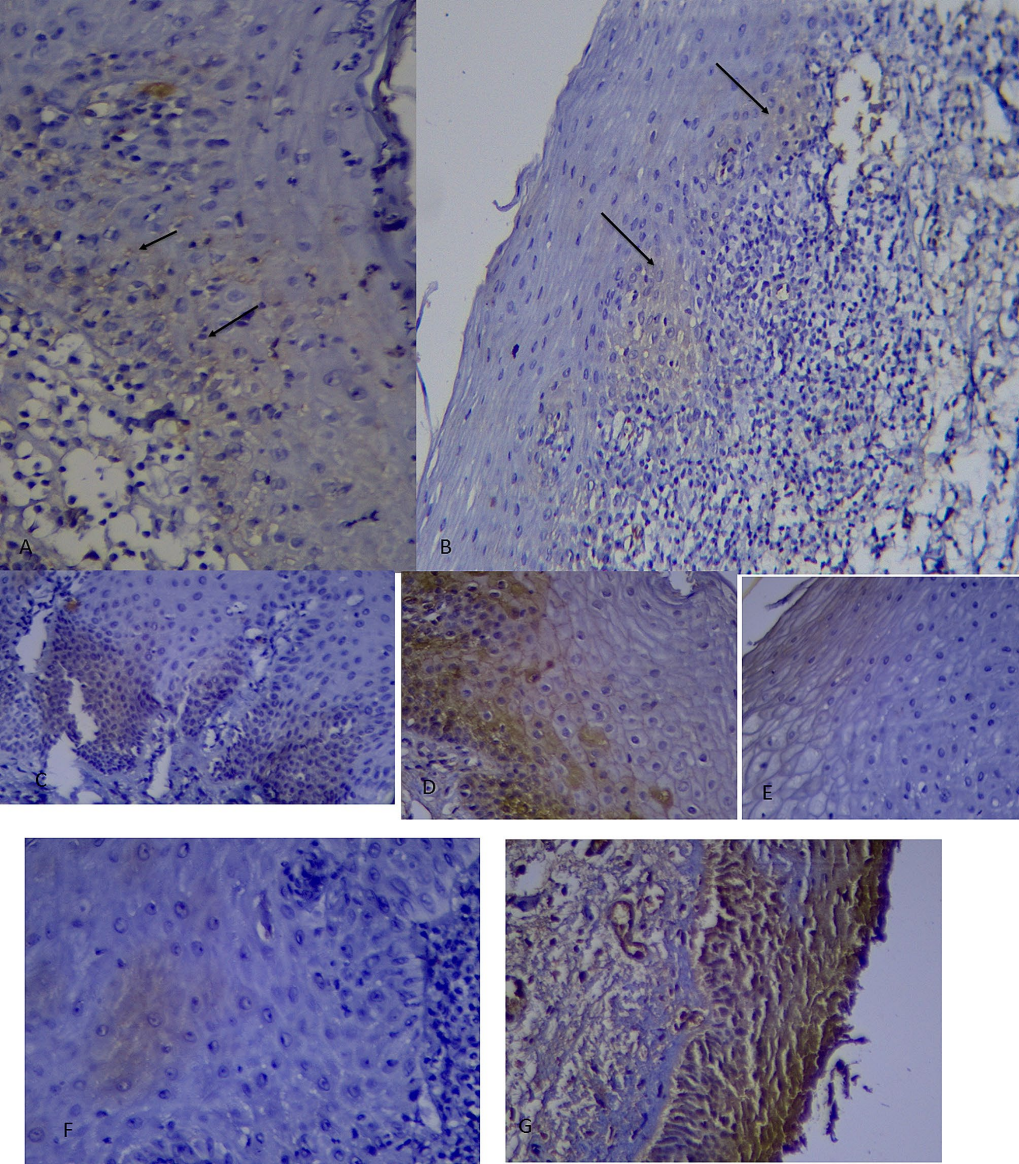
Photomicrograph shows, (**A**) Staining in basal-parabasal location in reticular group (400). (**B**) Staining in basal-parabasal location in erosive group (200). (**C**) Staining in basal-parabasal location (400) (**D**) Staining in basal-parabasal intermediate location (400) (**E**) Staining in upper intermediate location (400) (**F**) Staining in intermediate location (400) (**G**) Staining in all layer location (400)

The number and percentage of samples in different groups of stained cells location showed in Table 3. Kruskal-Wallis test showed that there was no significant difference between the location of stained cells in different layers of the epithelium in the 3 groups (*P*-value = 0.90) (Fig. 4).

**Table 3:** Location of stained cells in three groups

Location of stained cells		Reticular OLP (*n* = 15)	Erosive OLP (*n* = 16)	Control (*n* = 8)	*P*-Value
Basal-Parabasal	number percentage	5 33.33	6 37.50	3 37.5	0.90
Basal-Parabasal-Intermediate	number percentage	5 33.33	5 31.25	3 37.5	
Upper intermediate	number percentage	2 13.33	0 0	1 12.5	
Intermediate	number percentage	2 13.33	2 12.50	1 12.5	
All layers	number percentage	1 6.67	3 18.75	0 0	

## Discussion

The results of this study showed that, there was a significant difference only between the percentage of stained cells in erosive OLP and the control group. However, there was no significant difference in the mean percentage of stained cells between erosive OLP and reticular OLP and between reticular OLP and control.

From the control group to the erosive lichen planus, there is an ascending trend in EGFR staining. So, it probably shows that the malignant changes might increase in erosive type. According to the researchers’ opinion, clinician should emphasize on erosive type and consider the malignant change for it.

In 2012, Zhao et al. [28] described that there were significant differences in the expression of EGFR between the OLP with erosive and ulcerative lesions and without erosive and ulcerative lesions. Strongly positive rates of EGFR were seen in erosive and ulcerative OLP.

On the other hand, Cortés-Ramírez et al. [27] in 2014 reported that the EGFR is not related to any of the specific clinical and histopathological aspects of the OLP, and they suggested that more complex and different molecular mechanisms are involved in the process.

In 2015, Kouhsoltani et al. [29] showed that the lack of Her-2/neu (as a protein of EGFR family) overexpression indicates that molecular targeting of Her-2/neu protein is not recommended as adjuvant therapy in OSCC and OLP.

The present study showed that there was no significant difference between the frequency distribution of staining patterns in 3 types of lesions. In Cortés-Ramírez et al. [27] study in 2014, which was conducted on different types of OLP, most of the samples had membrane-cytoplasmic staining. In contrast, in present study, the samples which have the typical characteristics of OLP showed a more cytoplasmic staining pattern.

In the study by Kumagai et al. [22] in 2010, the occurrence of protein marker in the control group (normal mucosa) was observed more in the basal layer, while in the samples of OLP, all cases showed EGFR expression in basal and parabasal epithelial cells. Thirty-nine cases (88.6%) showed EGFR expression in the spinous layer and only in 5 (11.4%) cases reached the superficial layers. They also reported an increase in the expression of EGFR in keratinocyte cells of OLP lesions. In the present study, the occurrence of EGFR protein marker was seen in all layers and was not limited to basal and parabasal layers but there was no significant difference between the location of stained cells in different layers of the epithelium.

In a recent study by Ma et al. [18] in 2022, among 52 possible targets, TNF, IL-6, CD4, EGFR, IL1B, IL10, AKT1, VEGFA, TP53, and IL2 had the highest degree values, indicating that these targets are important in the development of OLP and are expected to be targeted for clinical treatment of OLP. They also recommended that Cordyceps sinensis as a traditional Chinese medicine could be a beneficial choice in the OLP treatment. The present study concluded that EGFR might probably utilized as a marker for the treatment for erosive type of lichen planus. Whereas there was no significant difference between EGFR expression in reticular OLP and erosive OLP and control group; therefore, EGFR is not applicable for the reticular type. Since reticular OLP is asymptomatic in most patients in comparison to erosive OLP which shows severe signs and symptoms, EGFR as an important treatment target for erosive type can be possibly noticed in this study.

González-Moles et al. [30] presented a hypothesis about the potential for the malignant transformation of OLP. In this scoping review, 20 systematic reviews and meta-analyses published until October 2022 were critically appraised. They recommended that OLP the potential for the malignant transformation hypothetically derives from the aggressions of the inflammatory infiltrate and a particular type of epithelial response based on increased epithelial proliferation, evasion of growth-suppressive signals, and lack of apoptosis.

Currently, the treatment of OLP is palliative. Patients with this disease commonly use adrenocorticosteroids and immunosuppressants to reduce inflammation and promote healing. However, OLP is prone to recurrence, and long-term hormone therapy has important side effects, such as mucosal atrophy, secondary candidiasis, and dryness [31, 32]. Therefore, finding medications without major adverse reactions is very crucial.

Various studies about EGFR expression in OLP lesions showed controversial results, however, it seems that this protein marker is associated with OLP. Therefore, further studies are recommended to clearly show this association and find efficient treatments for OLP. It is also suggested to carry out studies in which periodic follow-up of patients is done to check the incidence of oral cancer and malignancy.

## Conclusions

In conclusion, the results of this study showed that in comparison of reticular OLP, erosive OLP, and the control group there was a significant difference just between erosive OLP and the control group in the percentage of stained cells. There were no differences between these groups in pattern, intensity, and location of staining. So, the reticular lichen planus is as notable clinically as erosive type in terms of having the malignancy potential.

## Data Availability

The datasets used and/or analyzed during the current study are available from the corresponding author on reasonable request.

## Declarations

### Limitations of the study

The limitation of this study was the high cost of laboratory work and unavailability of demographic data of the patients.

## Abbreviations

OLP: Oral lichen planus
EGFR: Epithelial growth factor receptor
HER: Human epithelial receptor
OSCC: Oral squamous cell carcinoma
